# A Semi-Analytical Model to Predict Infusion Time and Reinforcement Thickness in VARTM and SCRIMP Processes

**DOI:** 10.3390/polym11010020

**Published:** 2018-12-24

**Authors:** Felice Rubino, Pierpaolo Carlone

**Affiliations:** Department of Industrial Engineering, University of Salerno, Via Giovanni Paolo II 132, 84084 Fisciano (SA), Italy; pcarlone@unisa.it

**Keywords:** liquid composite molding, vacuum assisted resin transfer molding, deformable porous medium, distribution media, preform compaction, process simulation

## Abstract

In liquid composite molding processes, such as resin transfer molding (RTM) and vacuum assisted resin transfer molding (VARTM), the resin is drawn through fiber preforms in a closed mold by an induced pressure gradient. Unlike the RTM, where a rigid mold is employed, in VARTM, a flexible bag is commonly used as the upper-half mold. In this case, fabric deformation can take place during the impregnation process as the resin pressure inside the preform changes, resulting in continuous variations of reinforcement thickness, porosity, and permeability. The proper approach to simulate the resin flow, therefore, requires coupling deformation and pressure field making the process modeling more complex and computationally demanding. The present work proposes an efficient methodology to add the effects of the preform compaction on the resin flow when a deformable porous media is considered. The developed methodology was also applied in the case of Seeman’s Composite Resin Infusion Molding Process (SCRIMP). Numerical outcomes highlighted that preform compaction significantly affects the resin flow and the filling time. In particular, the more compliant the preform, the more time is required to complete the impregnation. On the other hand, in the case of SCRIMP, the results pointed out that the resin flow is mainly ruled by the high permeability network.

## 1. Introduction

Advanced composites have encountered a widespread diffusion in different fields, from transportation to sporting goods as well as from energy to infrastructure and architecture, where the need for materials that are at same time stiff and light is constantly growing. Within the last few years, liquid composite molding (LCM) technologies significantly developed, becoming an effective candidate to high quality production compared with costly autoclave processing [1,2]. 

The concept behind the LCM process is to force catalyzed liquid resin to flow through a dry and opportunely shaped preform driven by positive and/or vacuum pressure. The resin flow is aimed at impregnating and saturating the reinforcing preform, filling the porosities and promoting an intimate contact between fiber and matrix. In vacuum-assisted resin transfer molding (VARTM), the fiber preform, plus some ancillary materials, is placed on a rigid die and hermetically covered by a flexible vacuum bag. Then, the pressure gradient is established within the bagged material by means of a vacuum pump promoting resin flow through the preform. Clearly, the operative pressure, given by the difference between the external atmospheric pressure and the applied vacuum pressure, is significantly lower than what commonly applicable in other LCM processes, namely resin transfer molding, high-pressure resin transfer molding, and RTM-light [3]. Since the injection system and the rigid counter-die are replaced, respectively, by significantly cheaper vacuum pump and vacuum bag [4], the VARTM process exhibits the significant advantage over other members of the LCM process family of an intriguing tradeoff between setup costs compression and high product quality. During the process, after the vacuum application, the bagged material is deformed in out-of-plane compression by the unbalanced external pressure experiencing a certain degree of compaction. As a consequence, the empty volume available for resin flow (preform porosity) as well as the local preform permeability decrease [4,5]. The incoming flow plays two distinct effects. On the one hand, it counterbalances the external atmospheric pressure reducing the pressure load acting on the fibrous preform. As a natural consequence, a relaxation of the preform is induced. On the other hand, the liquid resin at the flow front acts as a lubricant promoting a further compaction of the reinforcement [4]. Once the resin reaches the vacuum port, it is stored in a resin trap to avoid any damages to the vacuum pump. 

A rigid constraint to the infusion process planning is dictated by the gel time of the catalyzed resin system. Indeed, preform saturation should be imperatively achieved before the resin reaches the gel point; otherwise, the sharp viscosity increasing will prevent further resin advancement. In this regard, an effective strategy to reduce the infusion time relies on the usage of high permeability plastic networks, namely distribution media, which is placed above the nested reinforcement between the peel ply and the vacuum bag. This variant of VARTM is known as Seeman’s Composite Resin Infusion Molding Process (SCRIMP). The higher permeability of the flow enhancement medium favors the resin flow and reduce the infusion time. Indeed, the liquid resin easily flows through the distribution medium, mimicking a surface flow along the in-plane direction, and, afterwards, penetrates into the preform following a predominant transversal flow along the out of plane, i.e. through the thickness, direction. The distribution medium induces a through-thickness flow in the preform due to the significant difference in the permeability resulting in a three=dimensional flow front which, as above depicted, advances first through the distribution medium and after within the reinforcement [4,6]. This behavior has to be taken into account when the resin flow in the VARTM process is simulated increasing the difficulty in the modelling. 

Simultaneously to the modeling of the liquid flow and the variation of resin properties, when one deals with VARTM, the effect of textile compaction and laminate variable thickness have to be taken into account considering their direct implication for permeability, porosity and flow [4,7]. Due to the aforementioned reasons, in VARTM, the local thickness is evidently dependent on position of flow front (generally the distance from inlet). In addition, the fiber volume fraction can change as the applied pressure is redistributed between the resin and the preform. The effect of local fluid pressure and preform compressibility can be depicted as follows: the higher local fluid pressure at the inlet reduces compaction pressure on the reinforcement and increases thickness. Van Wyk [8] and Chen [9,10] first dealt with the compaction behavior describing both single and multilayer performs. Gutowsky [11] also proposed an equation to model the compaction effects of unidirectional lubricated fiber bundles. The model is the most commonly used and it was later extended to address the woven preforms [7]. In the case of SCRIMP process, the distribution medium determines the establishing of two different flow regimes: the flow through the free-fibers region (the distribution medium) and the fibers textile region (bulk porous medium). Therefore, the transverse flow (i.e. the out-of-plane flow through the thickness of the fiber preform) has to be considered. Preliminary studies of Sun et al. [12,13] led to a formulation of a 3D model flow which can handle the resin infusion from the high permeability medium to the fiber preform, however the model have high computational costs and the long times required do not match the industry requirements. Han et al. [14] proposed a hybrid 2^1/2^D and 3D flow models integrating the compressibility of fiber mats reinforcement. The simulated results obtained by the authors agreed with the experimental observations. Several works have been published dealing with the numerical simulation and the analytical closed form solution for the VARTM process [15,16,17,18]. Lopatnikov et al. [19] firstly derived the governing equations for the resin flow through a deformable porous media in VARTM to predict the flow front progression, the pressure fields, and the thickness variations. The authors assumed that the preform is an elastic material and the elastic modulus remains constant during the preform deformation, which allows them to relate the applied pressure to the preform deformation. Correia et al. [4] and Park et al. [20] also developed analytical models to solve the resin flow problem in case of vacuum-assisted resin infusion and resin film infusion processes. The authors also evaluated the influence of preform compressibility on filling time and thickness variation. However, they omitted the viscoelastic behaviors of wetted fibers due to the difficulty to implement the hysteresis effect in the preform compaction-relaxation model. 

In this paper, the effect of the preform compaction on the impregnation time was assessed integrating an analytical model, which simulates the fibers deformation according to the actual value of pressure applied on the material and the compliance of the preform, as a subroutine in a fluid-dynamics model solved in Ansys CFX suite. Different values of the preform stiffness were hypothesized. The compaction model and the coupled approach are compared with literature data in order to test its reliability. The analysis was performed simulating a simple VARTM process and the SCRIMP process adding the effect of the distribution medium on the reinforcement impregnation.

## 2. Numerical Model 

The velocity and pressure fields in LCM processes are commonly inferred modelling the fluid advancement as a flow through a porous medium, according to the well-known Darcy’s law, which, under the hypothesis of single scale porosity and permeability, writes

(1) $$v_{s}=-\frac{K}{\mu }\;\nabla p$$

Being ***v_s_*** the fluid superficial velocity (accounting for the volume porosity ϕ), ***K*** the permeability tensor, *μ* the resin viscosity and ∇*p* the pressure gradient. Equation (1) states the proportional relationship between the fluid flow rate and the acting pressure gradient. Assuming the liquid resin as incompressible, the set of equations to be solved is completed by the mass conservation

(2) ∇ v=0

In this work, viscosity changes, attributable for instance to temperature or degree of cure variations, were neglected and left for further studies. Indeed, in the VARTM the curing process is intentionally less rapid than the resin transfer molding (RTM) or the resin infusion (RI) processes. Therefore, the viscosity increasing, which could be an obstacle for or at least retard the resin flow, plays a less significant role compared to the preform compaction [21].

At each time instant during the impregnation process, the solution of Equations (1) and (2) provides the liquid pressure distribution. During the VARTM process, the liquid velocity in each location continuously changes due to first the variation in the distribution of pressure gradient and then to the permeability and porosity changes attributable to the compaction and relaxation of the preform. Defining as *P_a_* and *P_v_* the atmospheric and vacuum pressure, respectively, and as *P_f_* and *P_r_* the pressure load carried out by the fiber preform and the liquid resin pressure, the following balance equation subsists [22]:

(3) $$P_{a}-P_{v}=∆P=P_{f}+P_{r}$$

Equation (3) simply states that a certain amount of the actual pressure Δ*P* acting on the vacuum bag, depending on the local liquid pressure, is transferred to and balanced by the porous preform leading to its compaction or relaxation. 

**Figure 1:**
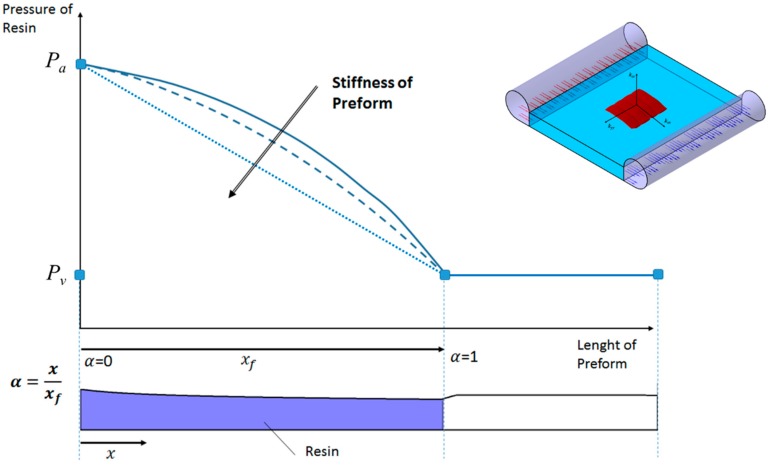
Resin pressure profile in a unidirectional impregnation test.

Obviously, the liquid pressure field is transient in nature and also dependent on the particular boundary conditions. Assuming, at this step of the analysis, that the resin flow is unidirectional along the *x* direction $\left(\bar{v}\approx v_{x}\right)$, one distributed inlet port and vacuum vent are applied and finally no distribution network is used in the process, the pressure profile follows the profile depicted in Figure 1.

Indicating as α the normalized abscissa ranging between the inlet port (α = 0) and the instantaneous flow front position (α = 1), for an infinitely stiff preform the pressure profile varies with α between *P_a_* and *P_v_* in a linear fashion. On the other hand, in the case of a preform characterized by finite stiffness, the pressure profile diverges from linearity and a closed form solution is not available. In addition, this behavior is more evident as the compliance (i.e. the inverse of the stiffness) of the preform increases [7].

The preform compaction (due to the vacuum application) and relaxation (strictly related to resin advancement and pressure redistribution) also influence the flow rate [20,23,24]. Indeed, the applied compacting pressure leads to the deformation of the preform mainly along the thickness direction. The reduction in thickness of the preform implies a reduction of porosity (which is related to the superficial velocity) and permeability [4,7]. The one-dimensional nature of deformation is commonly accepted and therefore the in plane strain components are typically negligible [10]. Under these accepted assumptions, the out-plane strain (i.e. preform thickness reduction) can be related to the global reduction of the preform volume during the compaction-relaxation cycle. Therefore, according to the analytical formulation proposed by Chen at al. [9,10] and assuming that the compaction is mainly due to the pores compression and the fiber volume of the preform is constant, a relationship between the fiber volume fraction and the preform deformation can be written as follows:

(4) $$\varepsilon \approx \frac{h-h_{0}}{h_{0}}=\frac{h}{h_{0}}-1=\frac{V_{f0}}{V_{f}}-1$$

where *h* and *h*_0_ are, respectively, the local instantaneous and initial preform thickness and *V_f_* and *V*_*f*0_ the instantaneous and initial (relaxed) local fiber volume fraction, respectively. Several models describing the porosity and volume fraction variations as a function of the compacting pressure *P_f_* are available in literature, starting from the pioneer work by Van Wyk [8], later extended by Robitaille [25,26,27] and Toll [28]. In the present analysis, the Toll model for unidirectional fibers perform [28,29] was assumed, considering the minor input parameters needed, if compared to the Robitaille model. According to this model, the local fiber volume fraction can be written following the following form:

(5) $$V_{f}={\left(\frac{P_{c}}{qE}+V_{f0}^{n}\right)}^{\frac{1}{n}}$$

where *q* is a preform dependent constant, *E* the Young modulus [8,28], and *n* a non-dimensional parameter. In particular, *q* is related to fiber properties, distribution architecture, and loading conditions, while *n* describes the reinforcement behavior in compression and depends on the properties of the single fiber tow. Combining Equations (4) and (5), one finally has

(6) $$\varepsilon =\frac{V_{f0}}{{\left(\frac{P_{c}}{qE}+V_{f0}^{n}\right)}^{\frac{1}{n}}}-1$$

Equations (5) and (6) suggest that preform deformation, fiber volume fraction, and permeability could change during the infusion stage depending on the applied pressure and the stiffness of the preform, summarized in the global parameters *qE*. Correia et al. [4] reported that different values in compliance can be obtained varying, besides the fiber material, the number of layers and the initial volume fraction when the preform is unloaded. The authors also pointed out that the inlet and outlet pressures applied are also a relevant factor in the curvature of the pressure field. Furthermore, from the fiber volume fraction, the longitudinal and transverse permeability can also be inferred, for instance using the Kozeny-Carman [30,31] or Gebart [32] models, to cite but a few. 

**Figure 2:**
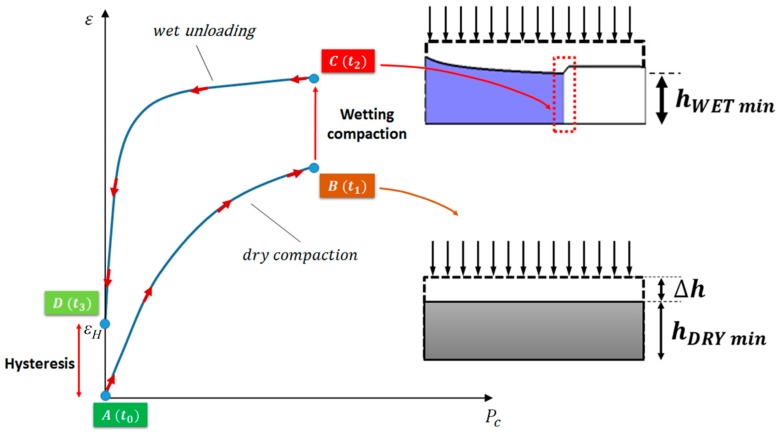
Loading (A→B)–unloading (C→D) curve of a generic reinforcement preform, highlighting wetting compaction (B→C) and hysteresis.

Previous studies demonstrated that the unloading curve of the preform, due to the resin front advancement and preform relaxation, does not exactly match the path followed at the loading stage. This is attributable to two main phenomena, namely the wetting compaction and the preform hysteresis [33]. Wetting compaction is a deformation effect observed at the arrival of the flow front, where the liquid resin plays a lubrication effect promoting a further nesting of the fiber tows and increasing the fiber–fiber contact points. Consequently, the wetted material experiences a higher degree of compaction with respect to a dry one even without variations in the applied pressure [34]. Therefore, once the fibers are fully impregnated and the resin pressure balance the external pressure determining the unloading of the fibrous preform a residual deformation is still present and the preform does not recover the initial thickness (“preform hysteresis”). In Figure 2, a qualitative loading-unloading curve for a fibrous preform is depicted, evidencing the wetting compaction and the preform hysteresis.

Wetting compaction and hysteresis are taken into account in the proposed model considering distinct calibrations of Equations (5) and (6), depending on the position along the A→B→C→D curve. In particular, the wetting compaction effect is mimicked introducing a discontinuity in the value of the n parameter, resulting *n_wet_* > *n_dry_*_._ The increase in this parameter is still dependent on the fiber type and preform architecture. Lundstrom [29], Sandlund [35], and later Toll [28] claimed that *n* can vary from 7 to 16. Analytically, it writes

(7) $$\{\begin{array}{c} V_{f\;A-B}={\left(\frac{P_{c}}{qE}+V_{f0}^{n_{dry}}\right)}^{\frac{1}{n_{dry}}}\\ \varepsilon_{\;A-B}=\frac{V_{f0}}{V_{f\;A-B}}-1 \end{array}$$

for the loading path, and

(8) $$\{\begin{array}{c} V_{f\;C-D}={\left(\frac{P_{c}}{qE}+V_{f0H}^{n_{wet}}\right)}^{\frac{1}{n_{wet}}}\\ \varepsilon_{\;C-D}=\left(\frac{V_{f0H}}{V_{f\;C-D}}-1\right)+\varepsilon_{\;A-D} \end{array}$$

for the unloading path. In Equation (8) *V_f0H_* and *ε_A−D_* represent the fiber volume fraction and the residual strain due to the hysteresis of the preform after a compaction-relaxation cycle [34].

**Figure 3:**
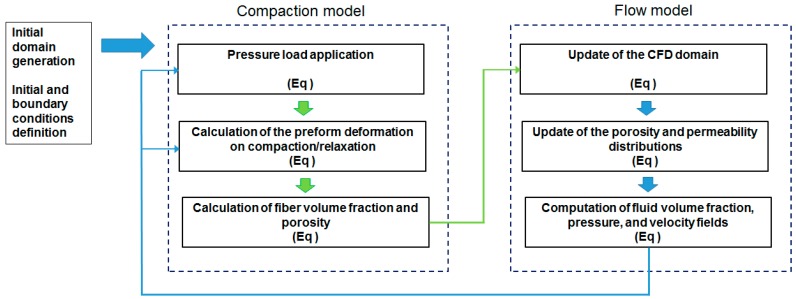
Schematic of the model.

The implemented model is based on the iterative solution of two different sub-models, namely a flow and a strain sub-model. In more details, the flow sub-model, which is basically a CFD model, computes the fluid volume fractions as well as pressure and velocity fields in the fluid phase. The strain sub-model describes the preform compaction and relaxation, allowing for the step by step updating of the CFD domain in terms of geometry, porosity, and permeability. Figure 3 reports a schematic depicting the main concept behind the full model. All models where implemented and solved using the ANSYS package by means of in-house developed subroutines.

**Figure 4:**
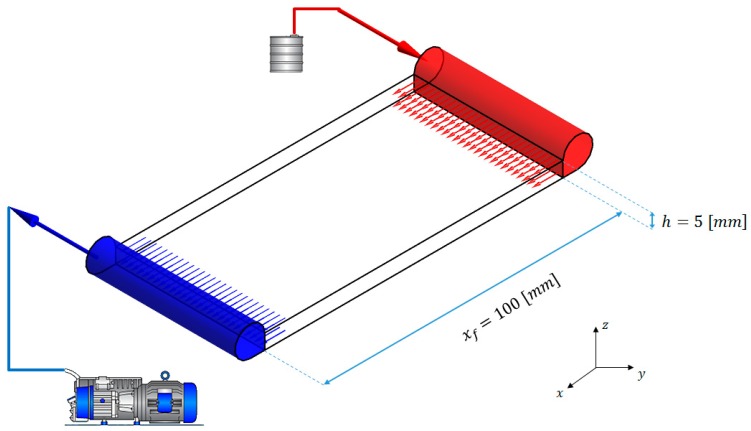
Simulated process.

The implemented model was applied to simulate the one-dimensional impregnation flow of a unidirectional single scale porous media, as depicted in Figure 4.

The length of the preform *x_f_* (in the main flow direction) was defined as 100 mm, while its thickness, in the initial relaxed configuration, was set at 5 mm. The following test cases, characterized by an increasing complexness, were simulated:

- Test 1: un-deformable preform without distribution network, assuming thickness, fiber volume fraction and permeability corresponding to a completely relaxed preform;
- Test 2: deformable preform without distribution network, considering variable thickness, fiber volume fraction and permeability as a function of the local compacting pressure;
- Test 3: un-deformable preform with distribution network, assuming thickness, fiber volume fraction and permeability corresponding to a completely relaxed preform;
- Test 4: deformable preform with distribution network, considering variable thickness, fiber volume fraction and permeability as a function of the local compacting pressure.

For Tests 2 and 4, three distinct values of stiffness of preform were taken into account to assess the influence of preform compliance on infusion time and thickness variation. The distribution medium, when contemplated, was modelled as an additional porous layer characterized by a 0.5 mm thickness (*h_DM_*), porosity and isotropic permeability (*K_DM_*) equal to 0.99 and 2.5 × 10^−9^ m^2^, respectively. A continuous flow condition was applied at the interface between the preform and the distribution network. In Test 2 and Test 4, i.e. when preform compaction and relaxation is allowed, the permeability distribution was updated at each simulation step following the Gebart model [32], which writes

(9) $$k_{xx}=k_{yy}=c_{1}\frac{{\left(1-V_{f}\right)}^{3}}{V_{f}^{2}}$$

(10) $$k_{zz}=c_{2}{\left(\sqrt{\frac{V_{f\;max}}{V_{f}}}-1\right)}^{\frac{5}{2}}$$

for the longitudinal and transverse direction, respectively. In the above equations, *c*_1_ (m^2^) and *c*_2_ (m^2^) are constant parameters depending on fiber radius and other features of the preform, while *V_f max_* is the maximum fiber volume fraction achievable which depends on the fiber packing. Obviously, both permeability components decrease when *V_f_* increases. Finally, taking into account that the out-of-plane stiffness of the distribution network is almost null, in Test 4 it was assumed that this element does not affect the compaction of the preform. Consequently, the thickness variation was analytically computed only for the nodes belonging to the reinforcing preform, assuming the thickness of the distribution media as constant during the process. The parameters used in the simulated cases are listed in Table 1.

The three distinct values of the stiffness parameter, *qE*, are hypothesized equal to, twice and eight times the value listed in the Table 1.

## 3. Results and Discussion

### 3.1. VARTM Process

Before analyzing the evolution of the flow front, the proposed model was validated with respect to the assessment of the prediction of the preform compaction by comparison with the outcomes provided by Grimsley in [34]. Obviously, simulations were run adopting the same material parameters, as indicated in Table 1, giving a thickness and fiber volume fraction equal to 3.8 mm and 0.52 at the end of the compaction step and 3.3 and 0.598 at the end of the relaxation step. The Grimsley’s model provides the following values: 3.2 and 0.513 at the end of the compaction step, and 2.8 and 0.583 at the end of the relaxation step, meaning an absolute deviation between the two prediction of 1.3% and 2.5%. In the authors’ opinion, the outcomes, in terms of preform deformation on compaction and relaxation, of the implemented and reference models reasonably agree each other. Considering that porosity and permeability values are directly derived from this deformation by means of Equations (6), (9) and (10), it can be claimed that the model is capable to evaluate the evolution of these quantities during the process.

**Figure 5:**
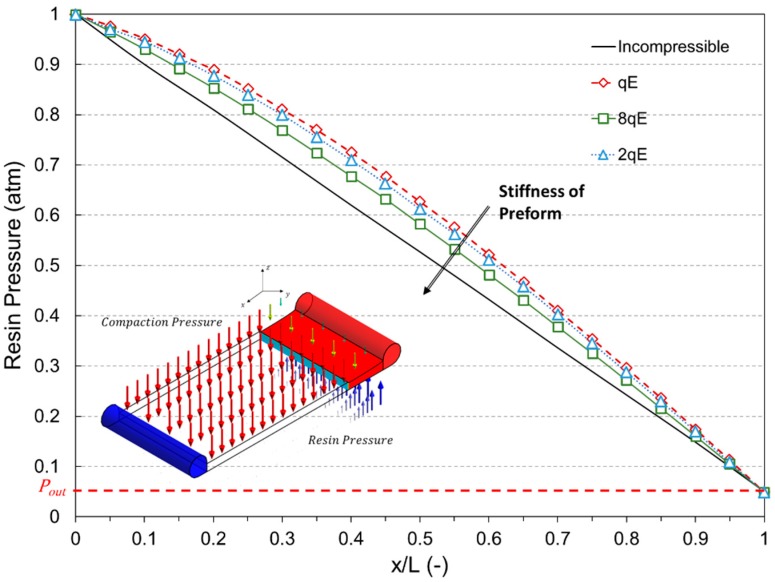
Pressure profile along the preform length at the end of impregnation.

The coupled mechanical-fluid model first provides the solution of the resin pressure field. Two main effects could be observed when a deformable preform is taken into account: the non-linear profile of pressure field and the variation of the laminate thickness. As shown in Figure 5, the resin pressure profile clearly deviates from the linearity exhibited in the case of rigid preform due to the increase in the compaction. The observed non-linear trends are related to an increase in compaction from the inlet to the vent, as confirmed by Correia et al. [4], which also determines the variation of permeability across the length of preform [7]. The departure from the linear profile increases with the increase of the preform compliance. In case of less compliant preform (the third case with stiffness equal to 8*qE*), the difference between pressure profiles is relatively narrow and remains below the 10% in almost all the field, suggesting that, in this case, the effects of the preform deformability are slightly significant [19].

**Figure 6:**
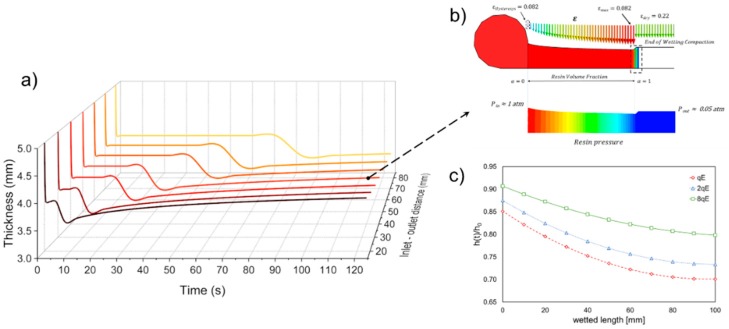
(**a**) preform thickness variation during the resin infusion in different locations between inlet and outlet (in case of preform stiffness equal to 289 MPa); (**b**) deformation and pressure distribution along the impregnation zone at the arrive of flow front in the middle of the preform length (i.e. 50 mm); (**c**) thickness variation form inlet to the flow front at the end of simulation as function of preform stiffness.

The variation in the final thickness of the laminate is very important from a manufacturing point of view being it strictly related to the dimensional tolerance of the final part. Furthermore, it can be used to predict the resin consumption and the local mechanical properties [4]. At the beginning of the process, the preform appears evenly compacted from the initial thickness of 5 mm to approximately 4 mm under the effect of the applied pressure gradient (see Figure 6a). This deformation is related to the first path of the loading curve, shown in Figure 2, representing the dry compaction effect. At the inlet section, the build-up in resin pressure induces the progressive unloading of the preform and its subsequent increase in thickness, porosity and permeability. At each location, a similar behavior of the preform can be observed: as the resin impregnates the fibers, the thickness further reduces due the lubricant action of the resin (see Figure 6b). This phenomenon is known as the wetting compaction (vertical segment of deformation profile in Figure 2). The minimum thickness, computed at the flow front due to the wetting compaction, identifies the point of maximum preform compaction, characterized also by the minimum permeability and porosity. After that, the thickness starts to increase as the compaction pressure reduces over time (wet unloading curve in Figure 2) [33,36]. When the resin pressure at a specific location approaches the atmospheric value the external compaction pressure is balanced and then unloading the preform, according to Equation (3) [22]. However, the initial thickness is not recovered and usually a residual deformation subsists in the preform even due to the preform hysteresis (point D of the deformation profile in Figure 2). The capability of the preform to recover the initial thickness is strictly related to its compliance: stiffest preform (green curve in Figure 6c) is characterized by a less pronounced deformation during the loading path and achieves a smaller dry compaction than the other two cases (see Table 2). Therefore, as the resin filled the preform in a specific location the preform recovers more than 90% of its initial thickness. On the other hand, the more compliant preforms (red and blue curves in Figure 6c) approached the 85% and 87% of the initial thickness at the inlet section, respectively.

**Figure 7:**
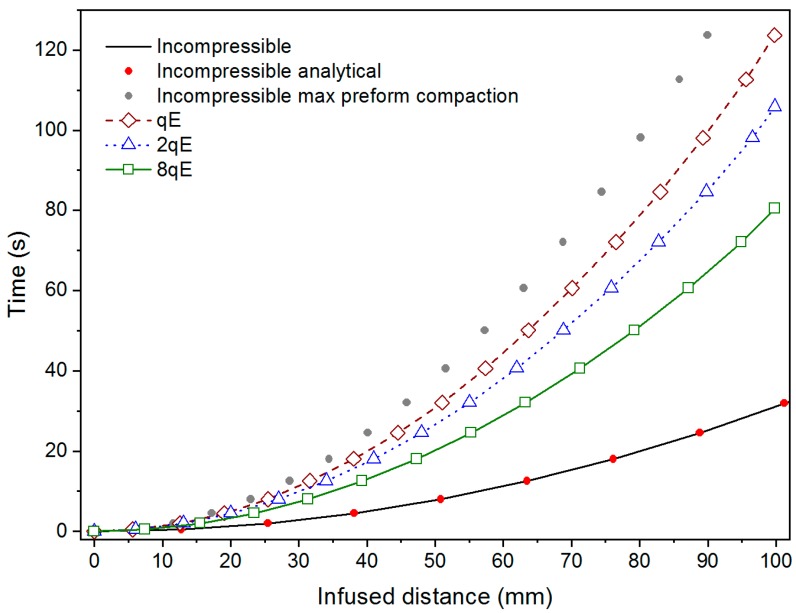
Infusion time profiles in Tests 1 and 2 for different values of the preform stiffness: numerical predictions and reference results.

As expected, the pressure gradient at the flow front reduces as a relatively stiffer preform is considered suggesting a potential lower flow rate. However, simulation outcomes indicate an opposite behavior, since the infusion time increases with the compliance of the preform (see Figure 7). This result is motivated by the fact that the synergic effect played by the increase in pressure gradient and reduction of the available volume to be filled by the fluid is not enough to compensate for the permeability reduction due to compaction. 

Figure 7 shows the infusion times computed in Test 1 and Test 2 (non-deformable and deformable preform, respectively) for the different values of the preform stiffness. In the interest of comparison and with validation purpose, numerical prediction regarding the flow though the uncompressible preform is also compared with reference results. They were inferred by means of the closed form solution of Darcy’s law (1) for unidirectional flow through and non-deformable single scale porous media (red dots in Figure 5). Analytically the infusion time writes as follow [4]:

(11) $$t_{inc}=\frac{\mu \;\left(1-V_{f\;inC}\right)x_{f}^{2}}{2\;∆P\;k_{xx\;inc}}$$

where *k*_xx_ is the permeability along the resin flow direction, evaluated using Equation (9).

The results of Test 2 are compared with the numerical outcomes of two distinct sub-cases for Test 1 (un-deformable preform without distribution network): (i) initial condition without compaction and (ii) fully compaction, in which the thickness of incompressible preform is assumed equal to the minimum thickness registered in the Test 2. The two scenarios were simulated, assuming preform thickness, fiber volume fraction, and permeability as 5 mm, 0.40, and 1 × 10^−10^ m^2^ (see Table 1) and 3.4 mm, 0.58, and 1.63 × 10^−11^ m^2^, respectively (solid black line and grey dots in Figure 5).

The reliability of the numerical prediction is apparent from Figure 7, being the theoretical and numerical curve, relative to the incompressible preform, practically overlapping. Observing the infusion time profiles for the two test cases, it can be argued that the changes in the volume fraction (and consequently in porosity and permeability) due to the finite stiffness of the preform and the decreasing thickness play a key role on the flow front evolution. Indeed, even if the empty volume to be filled by the liquid resin is relatively minor due to the increasing in fibers volume fraction and reduction in porosity, and the local pressure gradient is higher (see Figure 1) with respect to the un-compacted subcase, the increased flow resistance, which is related to the decreasing in preform permeability, implies a reduction in the velocity field at the front. Therefore, the resin requires more time to reach the vent and completely impregnate the preform. 

As can be seen, the hypothesis of deformable preform (Test 2) leads to an intermediate flow evolution between the un-compacted and fully compacted subcases of Test 1. Indeed, due to the finite stiffness of the preform and its variation in thickness, the local volume fraction the porosity and the permeability change continuously during the evolution of the flow front and pressure field redistribution. In this case, the expression of pressure field resulting from the combination of continuity equation (Equation (2)) and the Darcy’s law (Equation (1)), $d^{2}P/dx^{2}=0$, which refers to a linear profile of pressure in the domain, results invalid. Consequently, the solving equation for the fill time become unreliable. The ratio between the flow front position in the incompressible case and for compressible preform does not depend on time, but it remains constant during the infusion. It relies on the compliance of the matrix and on the initial porosity value (as reported also by Lopatnikov et al. [19]). Indeed, is equal to 0.63, 0.53, and 0.5 for 8qE, 2qE and qE stiffness preform cases, respectively. As for the un-deformable case, also for the Test 2 a validated analytical model was used to compare the results of the proposed approach [4]. In the case of unidirectional flow with distributed inlet port and vacuum vent and without distribution medium (see Figure 4), assuming constant inlet and vacuum pressure, integrating the Darcy model, it yields the following expression for the infusion time in case of the deformable preform:

(12) $$t_{def}=\frac{\mu \;x_{f}^{2}}{2{\left(\frac{k_{xx}}{\left(1-V_{f}\right)}\frac{dP}{d\alpha }\right)|}_{\alpha =1}}$$

Defining as *C_α_* the ratio between the pressure gradient at the flow front for the compressible and uncompressible preform cases [4],

(13) $$C_{\alpha }=\frac{\frac{dP}{d\alpha }}{∆P}$$

Combining Equations (11), (12) and (9) yields the following writing for the infusion time of a flexible preform,

(14) $$t_{def}=\frac{\mu \;x_{f}^{2}}{2\;c_{1}\;∆P}\;\frac{1}{C_{\alpha }}\;{\left(\frac{V_{f\;C}}{1-V_{f\;C}}\right)}^{2}$$

where *V_fC_* is the fiber volume fraction at the flow front. The above equation, congruently with numerical predictions, highlights that, in case of deformable preform, infusion time is affected by three main factors: the fiber volume fraction, the longitudinal permeability and the pressure gradient at the flow front. In more details, the pressure gradient induces a higher flow rate with respect to the case of infinite stiffness preform; the increase in volume fraction affects the preform properties in terms of porosity and permeability. In this regard, *C_α_* represents a preform parameter assuming values greater than unity and increasing with preform flexibility.

**Figure 8:**
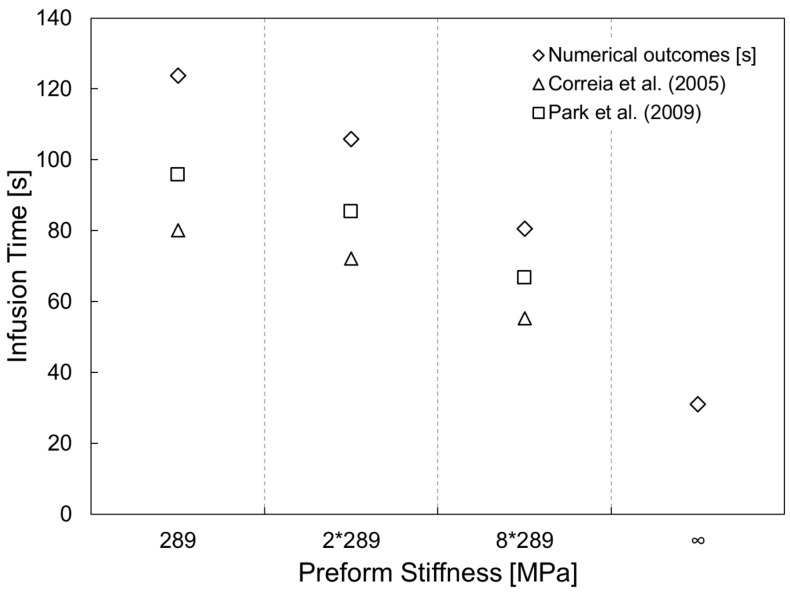
Fill time as function of preform stiffness.

Figure 8 shows the infusion time numerically calculated for different values of stiffness preform (*qE*), starting form high compressible reinforcement to a theoretical un-deformable preform. The numerical outcomes are compared with the analytical solution proposed by Correia et al. [4] and Park et al. [20].

The infusion time for a deformable preform is expected to be minor with respect to the non-deformable one, due to the minor volume of resin required to impregnate the preform. Conversely, the results numerical simulations describe that the infusion time decreases with the preform stiffness (Figure 8). It means that the minor volume to fill and the higher established pressure (see Figure 5) during the filling are not capable to balance the reduction of longitudinal permeability. In addition, as can be seen from the graph, the analytical models underestimate the time required for completing the infusion. The reason is that despite of it provided a good correlation with experimental results performed by the authors the model proposed by Correia and Park [4,20] neglected the wetting compaction effect due to the lubricant action of the resin. Conversely, it results more significant as the compliance of the preform increases resulting in a pronounced departure between the numerical and analytical results, as can be observed in Figure 8 for the first two cases, preform stiffness equal to 289 and 2 × 289 MPa, respectively. As the stiffness of the preform becomes bigger and bigger approaching the un-deformable cases, the discrepancy between the two results decreases pointing out a reducing relevance of the lubricant effect with respect to the preform stiffness. 

### 3.2. SCRIMP Process

**Figure 9:**
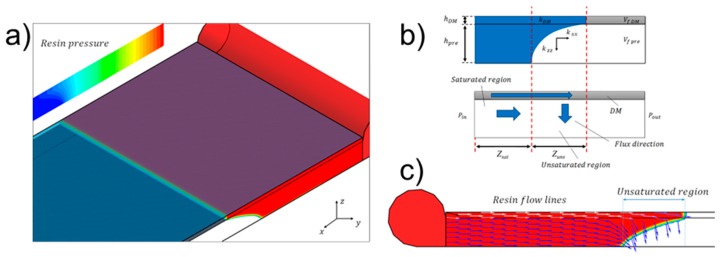
Flow evolution through the un-deformable preform with distribution network: (**a**) contour plot of resin volume fraction and pressure field during the infusion; (**b**) scheme of flow behavior; (**c**) resin volume fraction and resin velocity field (lateral view).

**Figure 10:**
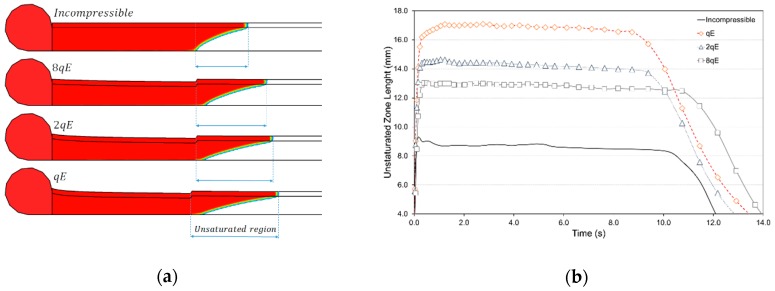
Effect of the preform stiffness on length of the unsaturated region during the infusion: (**a**) graphical representation with contour plot of resin pressure field; (**b**) unsaturated length profile against infusion time.

Test 3 and 4 simulated the infusion stage of the rigid and flexible preform, including also the distribution medium in the computational domain. Figure 9 and Figure 10 graphically show some of the obtained outcomes.

The main effect of the highly permeable distribution network is the establishing of two zones which dynamically evolve during the process: a through the thickness fully saturated zone, *Z_sat_*, where the fluid flows through the preform with a flat velocity profile and a through the thickness partially saturated zone, *Z_uns_*, characterized by longitudinal and transverse flows. In the former region the component of the velocity vector parallel to the main flow direction is, with reasonable approximation, the only non-null term, meaning that the flow is substantially unidirectional and no significant crossed flows are established. On the other hand, the flow in the latter zone is, for the considered case, bi-directional since the liquid resin permeates from the distribution network downstream toward the preform. As naturally expected, the velocity of liquid resin along the main flow direction is higher in the distribution medium due to the lower resistance to flow, which results in the complex shape of flow front observable in the unsaturated region.

As observable in Figure 9, as the unsaturated zone is approached, and the shape of the isobars significantly changes deviating from orthogonality to the preform length and assuming a curved shape according to the bi-dimensional flow. The observed behavior also verifies the hypothesis of constant flow front shape and linear pressure profile in the saturated region. What is more, in the saturated zone, the resin flowing through the distribution network exhibits higher velocity with respect to the resin in the corresponding layer of the partially saturated zone. This is related to the liquid mass transversally flowing from the upper layer toward the preform. From Figure 9, it can be observed that a small amount of liquid mass starts to transversally flow from the upper layer toward the preform also in the saturated region highlighting the occurrence of a transition region between saturated zone and flow front region. This transition results from the different flow dynamics conditions established in the two regions (main longitudinal flow with a negligible through-thickness flow in the saturated zone, while in the flow front one the transversal flow is dominant) and accounts for the balance of the total flow rate between the two regions [37,38].

Figure 10 shows the variation of the length of the un-saturated region as function of the preform stiffness. As observable in Figure 10b, the formation of the unsaturated region occurred as the infusion begins due to the high longitudinal permeability of the distribution medium compared to the reinforcement one. What is more, after a brief transition the unsaturated region length remained constant during all the infusion. The observed results confirmed the assumptions of Hsiao et al. [38] that the flow front region maintains its shape and advance with uniform velocity, which mainly relies on the thickness ratio between the distribution medium and the preform and the difference on their permeability. The length starts to drop as the flow front reaches the end of preform and tends to a zero value as the preform is progressively filled by the resin.

The following analytical solution was proposed by Fink et al. [38] for the case of un-deformable preform with distribution network:

(14) $$t_{inf,DM}=A_{1}Z_{sat}{}^{2}+A_{2}Z_{uns}$$

where *A*_1_ and *A*_2_ are constant values depending on the fluid and the materials structural parameters. In particular, these constants can be calculated starting from the fluid viscosity, the applied driving pressure, as well as porosity and permeability of distribution medium and preform [37,38].

**Figure 11:**
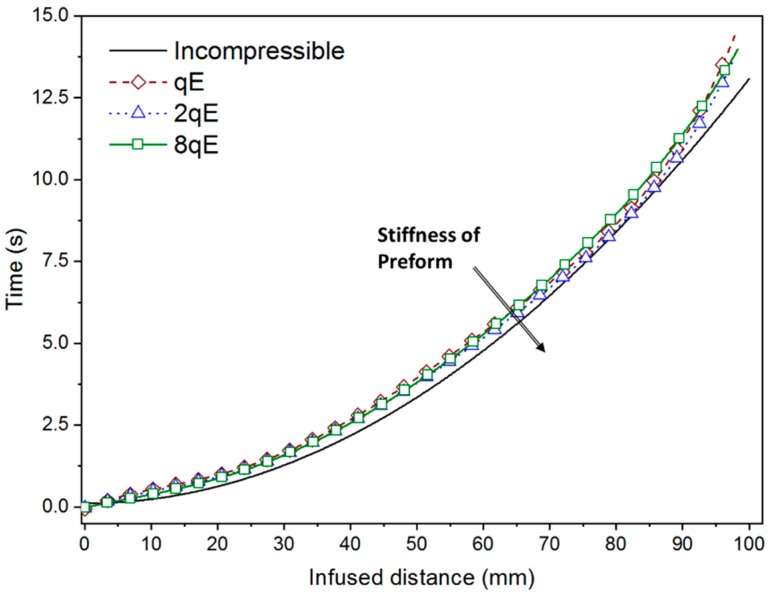
Fill time as function of infused distance for different values of the preform stiffness.

The infusion time in Test 3 was estimated as 11.6 s by the implemented model, against 12.4 s provided by the analytical solution. The prediction error, with respect to the closed form solution, resulted equal to 0.8 s (6%), so still acceptable. Figure 11 reports the fill time for different value of reinforcement stiffness plotted against the preform length.

**Figure 12:**
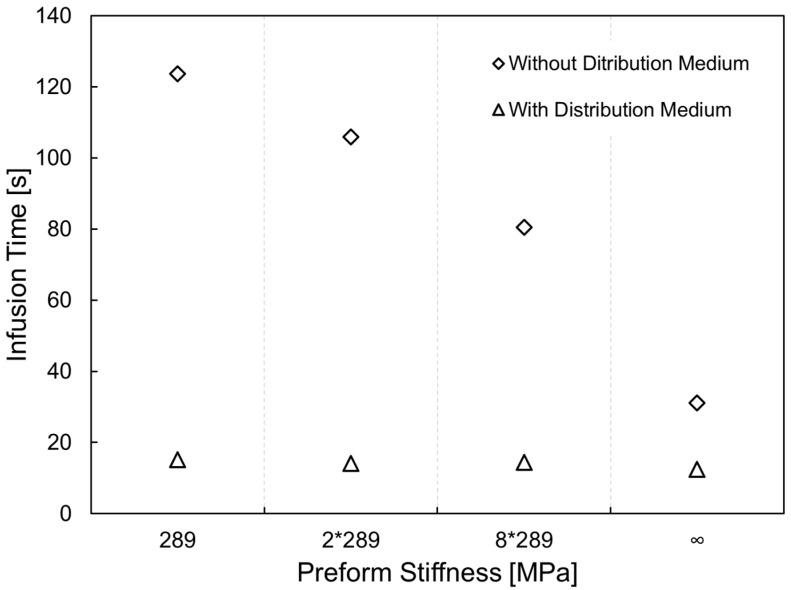
Time infusion comparison for vacuum assisted resin transfer molding (VARTM) and Seeman’s Composite Resin Infusion Molding Process (SCRIMP) processes as function of preform compliance.

Ac can be observed, no significant variations in the fill time occurred when the distribution medium is used for all considered cases. Indeed, the curves seem almost perfectly overlapping each other during the whole infusion, except for the incompressible case that slightly diverges from the other cases and requires a smaller time to complete the infusion (see Table 3). The reason of the observed behaviors can be related to the mutual changes in the process parameters, thickness, fibers volume fraction and permeability occurring during the process and affecting the flow of the resin. Previous studies [37,38] pointed out the competitive actions exerted by the mentioned parameters. Indeed, is was observed that as the thickness ratio between distribution medium and structural layer increases, due to the reduction of the preform thickness depending on the material compliance, the flow front velocity increases and the fill time consequently decreases. Moreover, the use of a network with higher permeability, more than two order of magnitude for high compliant preform, significantly affects the global flow rate of the resin. On the other hand, the reduction of longitudinal and transversal permeability of the structural layers in the range of values acquired during the infusion has a smaller effect compared to the distribution medium. Therefore, a global reduction in the fill time is expected for specific values of stiffness when the permeable network is used. Changes in the reinforcement volume fraction affects the resin flow, as observed for the Test 1 and Test 2. Indeed, as previously mentioned, when the fiber volume fraction raises the fill time is expected to increase due to the more relevant role played by the reduction in permeability than the opposite effect of the reduced volume that will be occupied by the resin. Being the impact of longitudinal and transverse permeability dominant on the porosity variation, it follows that congruently the length of the partially saturated region increases with the compliance of the preform. This last effect is also promoted by the pronounced transversal permeability reduction with respect to the longitudinal one (see Equations (7)–(10)). Numerical outcomes highlighted that these competitive actions balance each other determining a similar behavior of the resin flow and thus achieving comparable values of fill time (see Figure 11 and Figure 12).

The direct comparison between Test 1 and Test 3, assuming the same processing conditions, points out that for the considered setup the usage of the distribution network allows to achieve a time compression of about 62% (see Table 3). As can be observed from Table 3, the effect related to the presence of the distribution medium dramatically prevails on the impact related to the stiffness of the preform. In fact, in this case the higher flow rate is established in the top layer independently from the level of compaction and other properties of the preform. As a general observation, it can be argued that, when the stiffness of the preform increases, a relatively minor infusion time can be achieved, as is also observed in case of absence of distribution medium. This can be related to the fact that a less deformable preform tends to preserve its initial configuration, porosity and permeability if compared to a more flexible preform.

## 4. Conclusions

An integrated structural fluid dynamic approach was developed for the VARTM modeling. The proposed methodology couples an analytical model, which simulate the fibers preform compaction during the resin infusion, with the numerical model of resin flow. The only structural model was preliminary tested simulating the deformation along the thickness direction of a unidirectional fibers bed. The outcomes were compared with experimental data available in the literature to demonstrate the reliability of the model. Then, the integrated model was employed to simulate the one-dimensional infusion flow experiments. Different test conditions were taken into account: un-deformable preform that mimics the RTM process and three different compliant preforms. The lubricant action of the resin on the deformation behavior of the reinforcement was also considered. The numerical outcomes were compared with closed form solutions proposed by other authors. Obtained results pointed out that the higher is the compliance of the preform more time is required to complete the infusion. In case of the most compliant perform, stiffness equal to 289 MPa, the fill time was almost six times greater than the time evaluated in the un-deformable case. An increase in the fill time of approximately four and two times the un-deformable value was estimated for the middle tests, with stiffness equal to 2 × 289 and 8 × 289 MPa, respectively. The results highlighted that the reduction in preform permeability and, thus, the increase in flow resistance play a more relevant role on the resin infusion with respect to the increase in fiber volume fraction, which reduces the amount of resin required to completely fill the reinforcement. The developed methodology was also applied to the SCRIMP process. The model was first used to simulate the resin flow through the un-deformable preform. For validation purpose, the results were compared with analytical results available in literature. After, compliant reinforcements were considered in the simulations. As far the SCRIMP process, no significant variation in the process time were observed pointing out the high effect of the high permeable distribution medium on the resin flow regardless of the preform stiffness considered in the model. The model application presented in the paper suggests that it is able to provide reliable predictions of the resin flow and thickness development of the part and can be used to evaluate the infused resin volume as well as the dimensional tolerance. In addition, the results highlight that the compaction behavior may not even be characterized when a high permeable network is used in the VARTM process. 

## Figures and Tables

**Table 1:** Parameters used in the numerical simulations.

Parameter	Unit	Test 1	Test 2	Test 3	Test 4
*x_f_*	(mm)	100			
*H*_0_	(mm)	5			
*h_DM_*	(mm)	-		0.5	
*V*_*f*0_	-	0.40			
*V*_*f*0*H*_	-	0.46		0.46	
*V_fDM_*	-	-		0.01	
*c*_1_	(m^2^)	7.41e^−11^			
*c*_2_	(m^2^)	9.06e^−10^			
*K_DM_*	(m^2^)	-		2.5e^−9^	
*P_a_*	(Pa)	101325			
*P_v_*	(Pa)	5000			
*µ*	(Pa s)	0.1			
*qE*	(MPa)	∞	289	∞	289
*n_dry_*	-	-	11.85	-	11.85
*n_wet_*	-	-	15.45	-	15.45

**Table 2:** Thickness variation and deformation with the increase in preform stiffness.

Thickness/Deformation Values	*qE*	2*qE*	8*qE*
Initial thickness (mm)	5	5	5
Dry compaction thickness (mm)	3.91	4.13	4.54
*ε_dry_* (-)	0.21	0.18	0.09
Wetting compaction (mm)	3.4	3.6	3.95
*ε_wet_* (-)	0.32	0.28	0.21
Final thickness (mm) ^1^	4.25	4.37	4.53
*ε_hysteresis_* (-)	0.15	0.13	0.094

^1^ The final thickness is acquired at the inlet section, because at that location the equilibrium between compaction pressure and the pressure field inside the laminate has been reached and the hysteresis effect can be appreciated

**Table 3:** SCRIMP process simulation outcomes: numerically computed fill time for different values of preform stiffness and comparison with VARTM results.

Numerical Infusion Time (s)	*qE* (MPa)	Δ*t* Compared to Incompressible Preform (%)	Δ*t* between without DM and with DM	
			(s)	(%)
15.1	289	21.7	108.6	87.8
14.1	2 × 289	13.7	91.8	86.7
14.4	8 × 289	16.1	66.1	82.1
12.4	∞	-	18.7	60.1
